# Isolated Kimura Disease Presenting as a Brow Mass in a Saudi Male: A Rare Case Report

**DOI:** 10.7759/cureus.23000

**Published:** 2022-03-09

**Authors:** Askar K Alshaibani, Abdullah H Al-Mulla, Mazen A Mohammed, Mustafa Alhamoud, Sultan A Al-Hassan

**Affiliations:** 1 Ophthalmology, King Fahd Hospital of the University, Dammam, SAU; 2 Ophthalmology, Dhahran Eye Specialist Hospital, Dhahran, SAU; 3 Ophthalmology, King Khalid University, Abha, SAU

**Keywords:** soft tissue tumours, eyebrow mass, extra-orbital, mass, kimura disease

## Abstract

This is a case of a 36-year-old male not known to have any medical illness complaining of left upper painless swelling in the eyebrow with no systemic symptoms, and normal physical examination apart from the eyebrow mass. Computed tomography (CT) of the head showed a well-defined hyperdense subcutaneous soft tissue lesion seen at the medial aspect of the left orbit (eyelid-extra orbital). Excisional biopsy of the eyebrow mass was done and sent for histopathological evaluation which reports consistent findings with Kimura disease (KD) as a definitive diagnosis.

## Introduction

Kimura disease (KD) is defined as a chronic benign inflammatory condition associated with elevated eosinophil and high IgE levels; it mainly affects the subcutaneous tissue and lymph nodes [1]. It is usually found in the head or neck area as either a painless unilateral cervical lymphadenopathy or subcutaneous masses. The periauricular, axillary and inguinal lymph nodes and the parotid glands are commonly affected [2,3]. Kidney involvement is the most significant systemic manifestation of the disease, which can present in 10%-16% of KD patients [3]. Other systemic involvements are much less common.

The aetiology of the disease is not well understood, but it is proposed to be caused by an autoimmune or allergic mechanism [2,4,5]. Orbital KD is a rare manifestation of the disease, and the most commonly affected areas were the superior orbit, followed by the eyelids and then the lacrimal gland [5]. Orbital KD should not be confused with orbital malignant disorders such as orbital lymphoma and Kaposi’s sarcoma. In addition to clinical findings, the definitive diagnosis can be reached by imaging, biopsy, and histopathological examination, which play an important role in the management [6]. In this article, we discuss a rare presentation of KD that presented as an isolated brow mass in an adult Saudi male patient. 

## Case presentation

**Figure 1:**
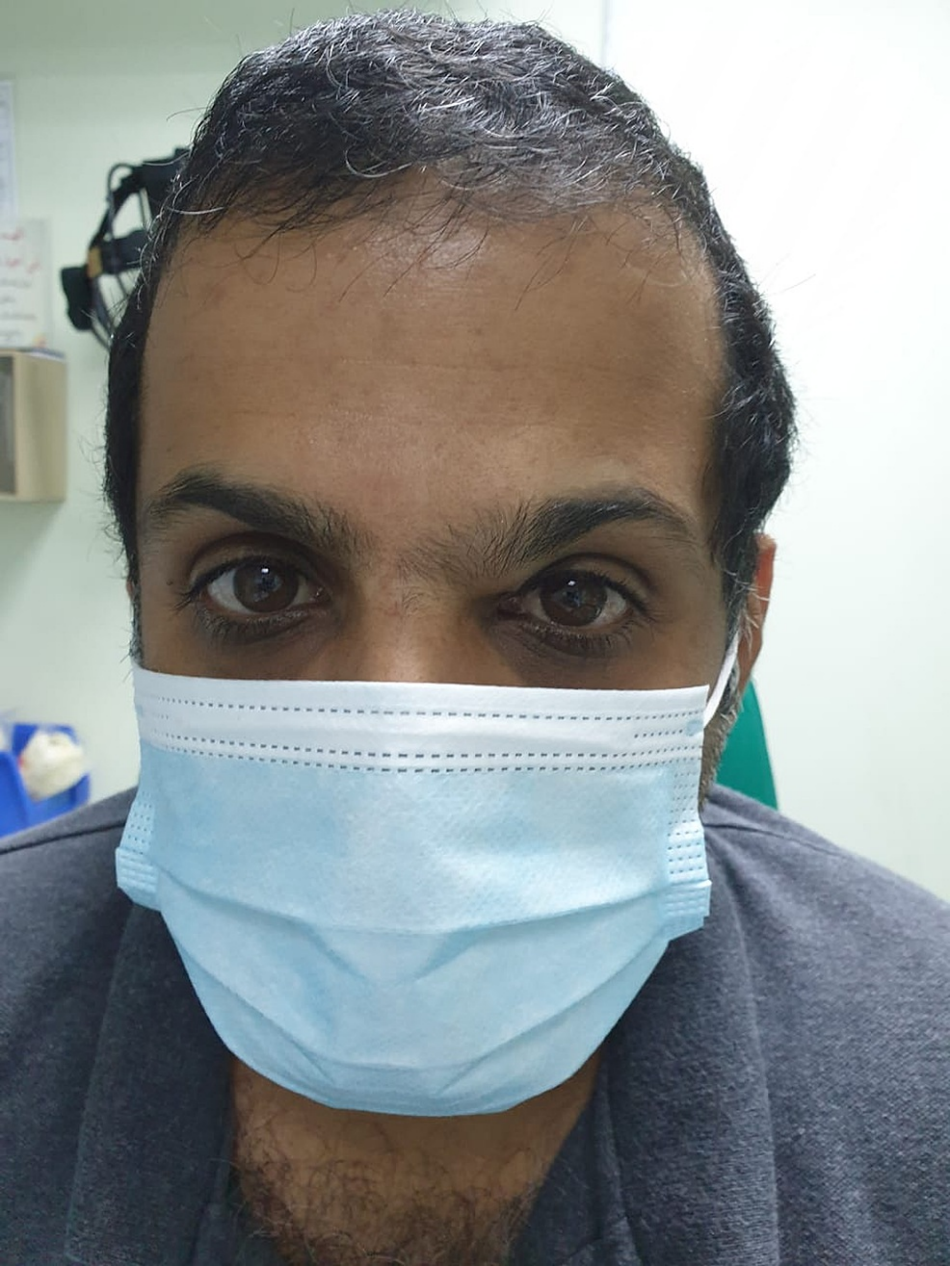
External front photo showing the brow mass.

**Figure 2:**
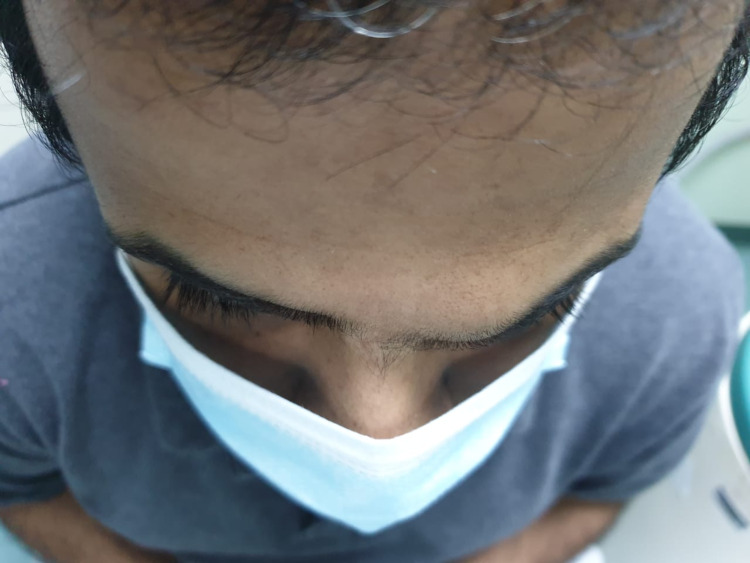
External superior view of the same mass.

A 36-year-old male not known to have any medical illness presented to the Oculoplastic clinic at Dhahran Eye Specialist Hospital in Dhahran, Saudi Arabia with a chief complaint of left upper swelling in the eyebrow. The swelling started three years prior to the presentation to the clinic, and it had increased in size during the previous four months. It was painless and non-tender, and the patient denied any systemic symptoms. On examination, there was a palpable mobile upper medial mass (1 x 2 cm) over the left eyebrow as illustrated in Figure 1 and Figure 2. There was no change in the skin color, discharge, or ulceration. Visual acuity was 20/20 in both eyes without correction. Extraocular muscle movements were not affected. Intraocular pressure measured by Goldman applanation was 15 mmHg and 12 mmHg of the right and left eyes, respectively. No afferent pupillary defect was detected. The anterior segment exam showed clear eyelids, quiet conjunctiva, clear cornea, deep and quiet anterior chamber, clear iris, and clear crystalline lens of both eyes.

**Figure 3:**
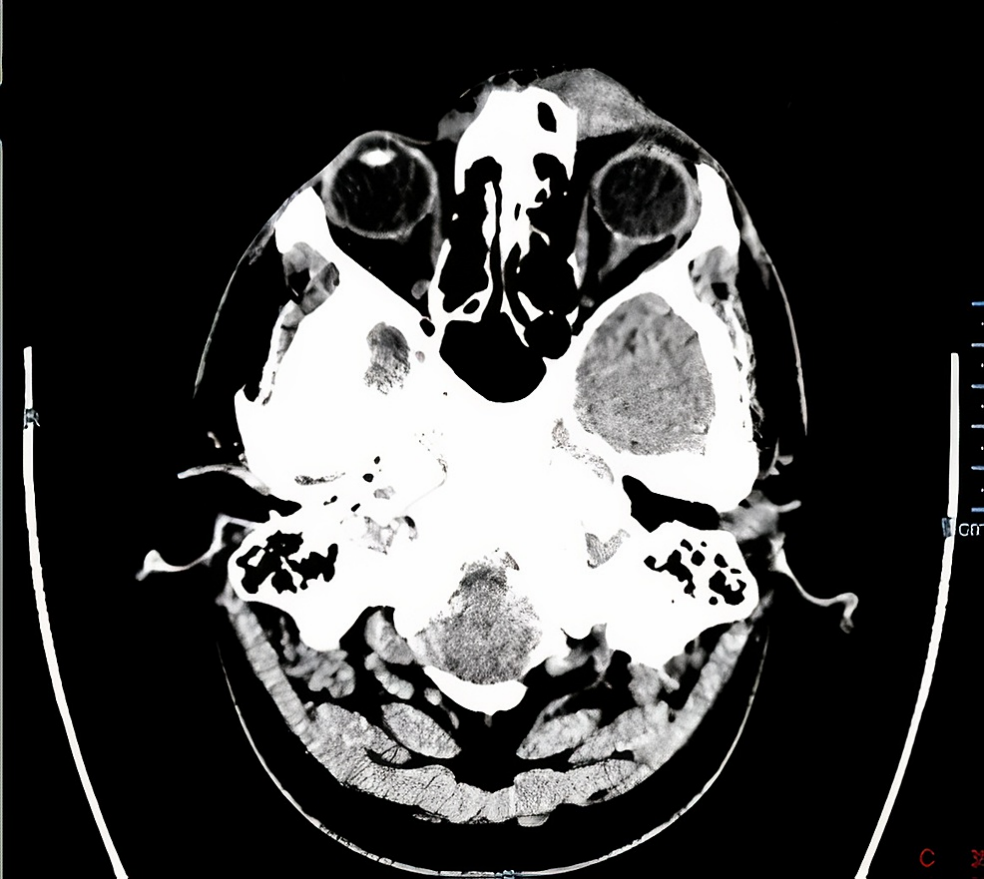
Computed tomography (CT) of the head with contrast showing a well-defined hyperdense subcutaneous soft tissue lesion seen at the medial aspect of the left orbit (eyelid-extra orbital).

**Figure 4:**
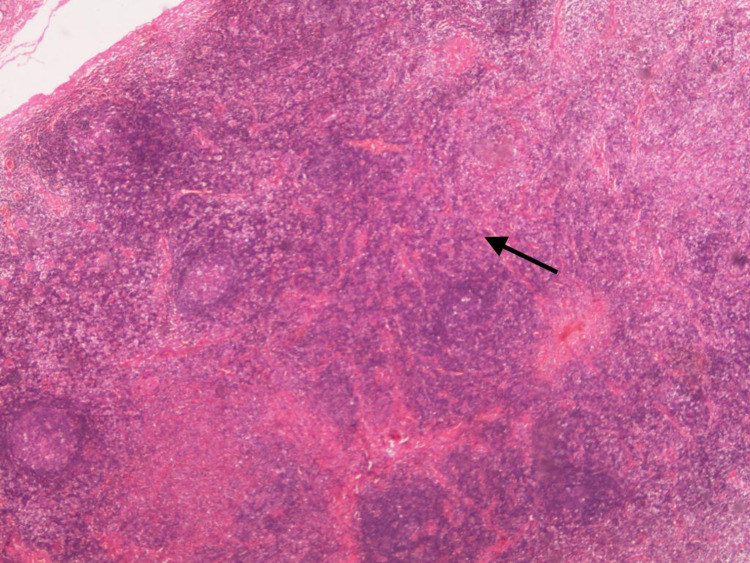
X40 magnification using Hematoxylin and eosin stain (H&E) which shows hyperplasia of the lymphoid tissue.

**Figure 5:**
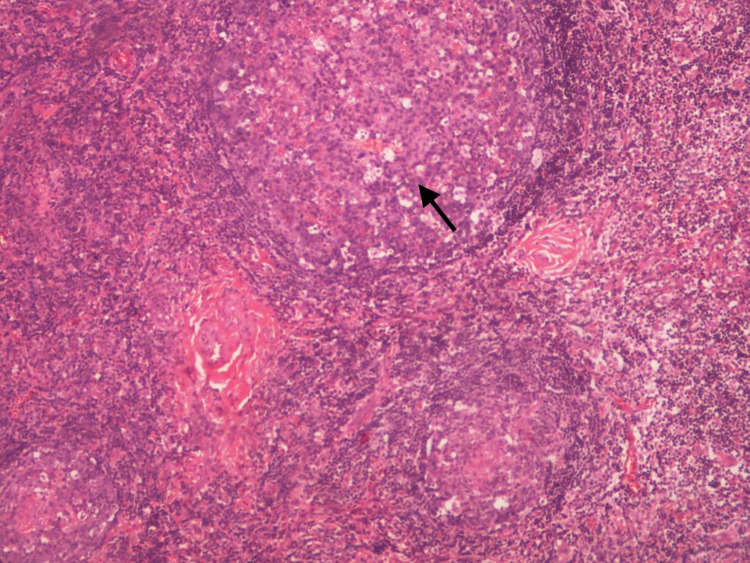
X100 magnification using Hematoxylin and eosin stain (H&E) which shows hyperplasia of the lymphoid follicles with germinal center fibrosis.

The dilated fundus exam showed clear vitreous, healthy discs, healthy macula, and unremarkable exam of the periphery. Computed tomography (CT) of the head with contrast was requested (Figure 3); it showed a well-defined hyperdense subcutaneous soft tissue lesion seen at the medial aspect of the left orbit (eyelid-extra orbital). There were no underlying bone changes or intracranial extension. After administration of the contrast, an avid enhancement was revealed. On CT, the mass measurement was 14.5 x 21 x 10.3 mm as anteroposterior, width, and height, respectively. Apart from these findings, the CT head scan was normal. The patient underwent an excisional biopsy of the mass under general anaesthesia. The specimen was sent for histopathological evaluation. The histopathology report revealed lymphoid tissue surrounded by muscle fibers. The lymphoid follicles showed germinal center hyperplasia, fibrosis, plasma cells, proteinaceous material in the germinal center, interfollicular eosinophils, eosinophilic abscesses, and focal proliferation of the thin-walled vessels. This histopathological report was consistent with KD as a definitive diagnosis. (Figures 4, 5). After multiple follow-up visits, there was no recurrence of the mass, and the outcome was satisfactory cosmetically and therapeutically for the patient.

## Discussion

KD is a chronic noncancerous inflammation of the soft tissues which is characterized by the presence of an elevation of eosinophils and IgE levels. Although the etiology of the disease is still not well understood, some authors have speculated that immune reactions to viral infections and some synchronous neoplasms might play a role in the disease development by the alteration of T-cell immunoregulatory functions [2,7].

KD mainly affects the subcutaneous tissues and lymph nodes, causing painless subcutaneous nodules, and it is usually associated with regional lymphadenopathy [1,4]. Moreover, KD was observed to have a predominance in the young Asian male population, and was also observed to coexist with nephrotic syndrome [2,6]. In most cases, KD is mild and has a self-limited course with gradual progression of the swelling. Sometimes, spontaneous regression is also achieved. In our case, the swelling started three years prior to his visit to the clinic and its size increased in the previous four months, which prompted the patient to seek medical assistance.

Some studies have addressed renal involvement of KD by the reported findings of renal biopsies. Ren et al. has reported that 59-78% of patients have nephrotic syndrome with mesangial proliferative glomerulonephritis and membranous nephropathy, as determined in the biopsy result, which is consistent with other reported studies [2,8]. However, in our case, a renal biopsy was not performed. Instead, our patient underwent an excisional biopsy of the mass, and it was sent for pathological examination. The histopathology report showed the presence of lymphoid follicles with germinal center hyperplasia, fibrosis, plasma cells, proteinaceous material in the germinal center, interfollicular eosinophils, and eosinophilic abscesses, and focal proliferation of the thin-walled vessels, which is consistent with KD.

There is no specific diagnostic feature of KD, which makes the diagnosis somewhat difficult. A differential diagnosis of KD can include a variety of inflammatory, neoplastic diseases, infections such as tuberculosis or toxoplasmosis, Kaposi's sarcoma, Hodgkin’s disease, dermatofibrosarcoma protuberans, and pyogenic granuloma, angiolymphoid hyperplasia with eosinophilia, among other conditions [4].

In terms of other diagnostic modalities, Deshpande et al. concluded that fine-needle aspiration cytology testing is a helpful preoperative diagnostic tool for KD in which the smear might show collagenous tissue with Warthin-Finkeldey polykaryocytes as well as a significant number of eosinophils and lymphocytes [9]. Since imaging studies could be diagnostic and can be helpful in both staging and assessing the progression of the disease with lymph node involvement, a head CT scan with contrast was requested for our patient. It showed a well-defined hyperdense subcutaneous soft tissue lesion at the medial aspect of the left orbit. There were no underlying bony changes or intracranial extension. After administration of the contrast, an avid enhancement was revealed. In our case, the diagnosis was made by integrating the clinical, histopathological, and radiological evidence.

Treatment options for KD include surgical excision, regional or systemic steroid therapy, and radiation; out of these, surgical resection is considered to be the first-line therapy, particularly with a localized mass [10], which was performed on our patient. Even though the recurrence rate after surgical resection is reported to be 25% [8], after multiple follow-up visits, there was no recurrence of the mass in our patient, and the outcome was satisfactory cosmetically and therapeutically.

## Conclusions

KD is a rare chronic inflammatory condition mainly affecting the subcutaneous tissue and lymph nodes with some systemic manifestations. The eyebrow is an unusual site of involvement for this disease. In this report, we presented a case of KD involving the eyebrow region. In summary, KD should be considered as one of the differential diagnoses in patients presenting with a brow mass. 
